# Emerging evidence linking stress and glucocorticoid signaling with cancer phenotypes

**DOI:** 10.1186/s12967-024-04962-w

**Published:** 2024-02-12

**Authors:** Anthony S. Zannas

**Affiliations:** 1 Department of Psychiatry, University of North Carolina, 438 Taylor Hall, 109 Mason Farm Rd, Chapel Hill, NC 27599-7096 USA; 2 Department of Genetics, University of North Carolina, 438 Taylor Hall, 109 Mason Farm Rd, Chapel Hill, NC 27599-7096 USA

**Keywords:** Cancer, Epigenetics, Glucocorticoids, Psychosocial stress

Stressful experiences have long been thought to contribute to cancer pathogenesis. Generations of physicians, dating as early as Galen, believed that tumors can result from mental distress, and literary characters have been portrayed to develop fatal tumors after experiencing chronic stress. These beliefs have been supported by epidemiological studies associating psychosocial stressors with certain cancers in humans and by experimental studies linking stress with tumorigenesis in animal models [1]. Among plausible underlying mechanisms, glucocorticoids are key molecular effectors of the stress response and have been studied extensively in cancer phenotypes. Such studies revealed a complex landscape, however, with glucocorticoid signaling exhibiting a wide range of either tumor-suppressive or oncogenic effects depending on cancer type and biological context.

While prior studies largely used high-dose or synthetic compounds to activate glucocorticoid signaling, less is known about how physiological levels of glucocorticoid activity influence cancer phenotypes. Addressing this question, a recent study showed that clear cell renal cell carcinoma (CCRCC) tissues are characterized by elevated expression of *NR3C1*, the gene encoding the human glucocorticoid receptor (GR) in humans [2]. *NR3C1* knockdown experiments further showed that reducing GR expression levels by 50% or more ameliorated cancer-related phenotypes, as shown by decreased cell proliferation and migration, and these effects were accompanied by reduced tumor size in cancer cell-injected mice [2]. These findings align with another recent study that modeled chronic stress in cell culture through prolonged exposure of human fibroblasts to the endogenous human glucocorticoid cortisol at physiological levels reached in human tissues during in vivo stress [3]. Prolonged cortisol exposure robustly increased fibroblast proliferation and migration, whereas these effects were abrogated when cells were co-treated with a selective GR antagonist [3]. Together these convergent studies spanning different model systems support GR downregulation or blockade as a promising molecular strategy for ameliorating cancer-related cell phenotypes.

What molecular events may underlie the impact of chronic stress and glucocorticoid signaling on cancer phenotypes? Among plausible mechanisms, epigenetics has emerged as a key molecular link between environment exposures and disease outcomes. Prolonged GR activation has been shown to induce functional changes in DNA methylation—a critical epigenetic modification in humans—that are widespread but preferentially affect genes involved in cell proliferation and migration [3], which are cell phenotypes relevant to cancer development and progression. Yet humans are constantly exposed to complex environments, and the effects of aberrant glucocorticoid signaling likely do not act in isolation but in concert with other cancer risk factors, such as smoking and diet that also influence the epigenome. Supporting this hypothesis, work in mice shows that restraint stress and cigarette smoke act in concert to promote colon tumor growth [4]. Beyond epigenetic regulation, aberrant GR signaling likely also influences a wide range of biological processes implicated in cancer pathogenesis. For instance, downregulating the GR through *NR3C1* knockdown was shown to activate endoplasmic reticulum stress and induce mitophagy in CCRCC cells, whereas directly inhibiting these processes counteracted the effect of GR downregulation on cell proliferation and migration [2]. Immune deregulation has also been recognized as a key link between stress and cancer, with potential pathogenic roles both within the tumor microenvironment and systemically. For example, chronic stress has been associated with immunosuppression that could in turn promote cancer development and progression by allowing malignant cells to evade immune surveillance. In line with this hypothesis, convergent experiments in 3D cell culture and mouse models show that stress and cortisol decrease immune cell infiltration in both spheroids and mammary tumors [5]. Taken together, such observations have begun to shed light on the multilayered mechanisms through which excessive stress and aberrant glucocorticoid signaling enhance tumorigenesis and impede efforts to improve outcomes.

Despite this emerging evidence, efforts to translate mechanistic insights to meaningful interventions are likely to face major challenges. The strongest evidence linking stress and cancer to date comes from cellular and animal models, whereas studies in humans have been less conclusive [1]. Such lack of convergence could result from several factors, including limitations in performing controlled experiments in humans, confounding by undocumented environmental exposures, inadequate modeling of potentially synergistic mechanisms, and vast phenotypic and molecular heterogeneity within single cancer diagnoses. To address these limitations, future studies need to employ innovative interdisciplinary approaches that integrate findings from translationally relevant model systems and human cohorts with well documented longitudinal stressors and cancer phenotypes. While stressful experiences are ubiquitous and often unavoidable, developing and targeting personalized interventions to vulnerable individuals will be essential for improving disease outcomes.

## Data Availability

Not applicable.
